# Allelic variation for alpha-Glucan Water Dikinase is associated with starch phosphate content in tetraploid potato

**DOI:** 10.1007/s11103-021-01236-7

**Published:** 2022-01-07

**Authors:** J. G. A. M. L. Uitdewilligen, A. M. A. Wolters, H. J. van Eck, R. G. F. Visser

**Affiliations:** 1 Plant Breeding, Wageningen University & Research, PO Box 386, 6700 AJ Wageningen, The Netherlands; 2 The Graduate School for Experimental Plant Sciences, Wageningen, The Netherlands; 3 Present Address: Limgroup BV, Born, The Netherlands; 4 Centre for BioSystems Genomics, Wageningen, The Netherlands

**Keywords:** Alpha-Glucan Water Dikinase, Association analysis, Phosphate content, Allelic diversity, *Solanum tuberosum*

## Abstract

**Key message:**

Association analysis resulted in the identification of specific *StGWD* alleles causing either an increase or decrease in starch phosphate content which was verified in diploid and tetraploid potato mapping populations.

**Abstract:**

Potatoes are grown for various purposes like French fries, table potatoes, crisps and for their starch. One of the most important aspects of potato starch is that it contains a high amount of phosphate ester groups which are considered to be important for providing improved functionalization after derivatization processes. Little is known about the variation in phosphate content as such in different potato varieties and thus we studied the genetic diversity for this trait. From other studies it was clear that the phosphate content is controlled by a quantitative trait locus (QTL) underlying the candidate gene α-Glucan Water Dikinase (*StGWD*) on chromosome 5. We performed direct amplicon sequencing of this gene by Sanger sequencing. Sequences of two *StGWD* amplicons from a global collection of 398 commercial cultivars and progenitor lines were used to identify 16 different haplotypes. By assigning tag SNPs to these haplotypes, each of the four alleles present in a cultivar could be deduced and linked to a phosphate content. A high value for intra-individual heterozygosity was observed (*Ho* = 0.765). The average number of different haplotypes per individual (*Ai*) was 3.1. Pedigree analysis confirmed that the haplotypes are identical-by-descent (IBD) and offered insight in the breeding history of elite potato germplasm. Haplotypes originating from introgression of wild potato accessions carrying resistance genes could be traced. Furthermore, association analysis resulted in the identification of specific *StGWD* alleles causing either an increase or decrease in starch phosphate content varying from 12 nmol PO_4_/mg starch to 38 nmol PO_4_/mg starch. These allele effects were verified in diploid and tetraploid mapping populations and offer possibilities to breed and select for this trait.

**Supplementary Information:**

The online version contains supplementary material available at 10.1007/s11103-021-01236-7.

## Introduction

Potato is a healthy and nutritious part of the average Western human diet, contributing carbohydrates and important amino acids and vitamins. It is, next to corn and wheat, one of the main sources of starch. Starch and its derivatives are widely employed in the manufacture of paper, textiles and adhesives, and due to their biodegradable and renewable nature they are increasingly being considered as an environmentally-friendly alternative to using synthetic additives in many other products, including plastics, detergents, pharmaceutical tablets, pesticides, cosmetics and even oil-drilling fluids (Kraak 1992). The thermal and rheological properties of potato starch, as well as properties in processing are related to the degree of starch phosphorylation (Veselovsky 1940). The presence of phosphate groups in starch increases the water-binding capacity, viscosity, transparency and freeze–thaw stability of processed potato starch (Craig et al. 1989; Swinkels 1985). Although natural starch from many plant species contains small amounts of covalently-bound phosphate, potato starch is particularly rich in phosphate (Jobling 2004). Phosphate groups are found attached to amylopectin chains at both C-3 and C-6 positions of the glucose residue (Bay-Smidt et al. 1994; Hizukuri et al. 1970; Sonnewald and Kossmann 2013). Several genes involved in starch biosynthesis and breakdown affect the level of starch-bound phosphate (Kötting et al. 2010; Carpenter et al. 2015) and can be proposed as candidate genes for starch phosphorylation. The most promising one is Glucan Water Dikinase (GWD1 or *StGWD*), a gene first described as the R-locus in potato (Lorberth et al. 1998). This single copy gene is a key enzyme in starch breakdown (EC 2.7.9.4) and catalyzes the transfer of phosphate to the C-6 position of glucosyl residues of the amylopectin fraction (Ritte et al. 2006; Zeeman et al. 2007). Genetic modification of plants overexpressing this gene permits the production of high-phosphate starch (Lorberth et al. 1998; Ritte et al. 2002). Likewise, silencing experiments have shown that potatoes with lower activity of this enzyme have a significantly lower level of starch-bound phosphate (Lorberth et al. 1998; Viksø-Nielsen et al. 2001; Wickramasinghe et al. 2009). The lower amount of starch-bound phosphate decreases starch degradation and sugar accumulation in potatoes during cold storage (Lorberth et al. 1998). Hence, a further reduction of phosphate in potato starch might contribute to potatoes with increased resistance to cold-sweetening. Breeding for a further increase of the phosphate content in potato starch on the other hand is highly desirable because a high natural degree of phosphorylation could lead to a higher substitution degree thus making chemical modification processes more efficient and potentially more environmentally friendly.

In the past two decades of potato research, identification of genes and markers that control the genetic variation of complex quantitative traits like starch phosphate content has mainly been done by linkage analysis in bi-parental segregating populations. These mapping populations are often developed from diploid parents that originate partly or completely from wild species. Such populations sample a maximum of four alleles in a single study and observed gene effects are often not representative of those found in elite tetraploid cultivars (Simko et al. 2004). In contrast to linkage mapping, association analysis samples a much larger number of alleles and usually cultivars with existing phenotypic information are used, representative for an elite genepool. Conceptually, there are two different approaches to identify DNA polymorphisms associated with quantitative trait loci (QTL) within an association analysis framework: a genome wide association analysis (GWAS) and a candidate gene approach. D'Hoop et al. (2008) has conducted an initial study to explore the potential of GWAS in potato by applying a genome-wide set of AFLP and SSR markers. In this study, a germplasm panel of 430 tetraploid potato cultivars was assembled and phenotyped. The panel covered a world-wide set of cultivars and progenitor lines, complemented by breeding lines, covering the entire range of commercial potato with respect to country of origin, year of release and market segment (consumption, frying and starch industry).

In a candidate gene-based association mapping approach, genotyping is targeted to functional and positional candidate genes for the trait under consideration. Background information on the physiology and biochemistry of the trait, together with knowledge on gene function from model organisms may suggest functional involvement of the candidate gene. Additional support may be provided by positional information of QTL from linkage maps or physical maps that locate a gene to the chromosome region suspected of being involved in the trait. In potato, candidate gene-based association mapping has been conducted by re-sequencing (Li et al. 2005; Simko et al. 2004; Wolters et al. 2010), by High Resolution Melting (HRM) analysis (de Koeyer et al. 2009) and by sequence based analysis using micro arrays or SNPs derived from Genotyping by Sequencing (GBS, Carpenter et al. 2015; Klaassen et al. 2019; Prodhomme et al. 2020; Rosyara et al. 2016).

Haase and Plate (1996) calculated a high heritability (*h*^*2*^ = 0.83) for starch-bound phosphate content in potato. Werij et al. (2012) confirmed *StGWD* as a candidate gene that underlies one of the three starch phosphate QTLs in the backcross diploid C × E mapping population of potato. The QTL analysis showed three major additive QTLs on chromosomes 2, 5 and 9, each explaining approximately 20% of the observed variance. In other studies these different chromosomes were identified to be important as well (Carpenter et al. 2015; van Harsselaar et al. 2017). The QTL on chromosome 5 co-localizes with the *StGWD* locus. The BC_1_ structure of the diploid C × E population however only allows the characterization of three GWD alleles in four possible combinations in the offspring. Thus, an unresolved question is how the level of starch phosphorylation is influenced by *StGWD* alleles in elite potato cultivars in which many more alleles, in different allelic compositions, are expected.

In this paper we investigate whether the collective information of quantitatively scored (i.e. dosage of) SNPs would enable us to deduce the composition of GWD haplotypes in individual tetraploid potato cultivars. We identified 16 distinct and highly diverse haplotypes and assigned tag SNPs to each of them. With this set of tag SNPs we were able to identify the fully informative four-allele GWD configurations for almost all of the nearly 400 sampled potato cultivars. The genetic composition of the cultivars was used to identify genotypic and allelic associations with starch-bound phosphate. We validated the marker-trait associations found in a diploid mapping population and show that one specific allele has a reducing effect on starch phosphate. We also identified a novel allele associated with an increase in starch phosphorylation, ready to be used in marker assisted breeding. We show that with a specific combination of four alleles a threefold higher level of phosphate content in starch could be achieved in tetraploid potato.

## Materials and methods

### Plant materials and DNA isolation

To aid haplotype identification five monoploid potato reference genotypes were used: 7322 (H7322, or AM79.7322 originally from G. Wenzel, Institut für Genetik, Grünbach, Germany), M47 and M133 (1022M-47 and 1022M-133) (Hoogkamp et al. 2000) and M5 and M38 (851-5 and 851-38) (Uijtewaal 1987). Furthermore, DNA from nine diploid reference genotypes was used: C and E (US-W5337.3 and 77.2102.37) (Hanneman Jr and Peloquin 1967; Jacobsen 1980), 1024-2 and 1029-31 (87.1024/2 and 87.1029/31) (Jacobsen et al. 1989), RH and SH (RH89-039-16 and SH82-93-488) (Rouppe Van Der Voort et al. 1997; Van Os et al. 2006), RH90 and RH88 (RH90-038-21 and RH88-025-50) (Park et al. 2005), and G254 (Olsder and Hermsen 1976). The cultivar collection used consisted of 430 tetraploid potato cultivars and progenitor lines chosen to represent a diverse range of commercial potato cultivars with respect to country of origin, year of release and market segment (D'hoop et al. 2008). Offspring plants of the diploid cross C × E as well as from the tetraploid cross cv. Astarte × cv. Voran and from a selfing population of cv. Sunrise were used for validation experiments. Genomic DNA was extracted from leaf material according to the protocol of van der Beek et al. (1992).

### StGWD gene sequence

As no genomic sequence of the potato GWD gene was available at the time we started our research we sequenced a full length genomic allele of the *StGWD* gene (Genebank JQ388473) from a BAC clone of the diploid RH89-039-16 genotype. The BAC clone (RH033J14, Genbank AC237986) was anchored to the ultra-dense SH × RH genetic map (Van Os et al. 2006) and located *StGWD* to BIN37 of the upper arm of chromosome 5, between markers GP179 (BIN27) and the centromere (BIN46) and at a 12 cM distance of the marker SPUD237 (BIN20) (De Jong et al. 1997) (data not shown). The 16.5-kb gene contains 34 exons and encodes 1464 amino acids. In the sequenced *Solanum phureja* DM whole genome assembly (Xu et al. 2011) the *StGWD* gene is located on superscaffold PGSC0003DMB000000248 of chromosome 5, and formerly annotated as gene PGSC0003DMG400007677 (iTAG transcript ID Sotub05g014130.1.1), recently as Soltu.DM.05G009520 (DM v6.1 annotation).

### PCR amplification and sequencing

Amplification and sequencing primers (Supplementary Table 1) were designed based on the consensus sequence of available genomic, mRNA and EST sequences and amplified both coding and non-coding sequence intervals of the *StGWD* gene. PCR amplicons for sequencing were generated from 50 ng genomic DNA template. Amplifications were performed in 20 μl reactions using 1 u of Taq Polymerase, 1 × reaction buffer, 200 nM dNTP and 250 nM of each primer. Standard cycling conditions were: 4 min initial denaturation at 94 °C, followed by 35 cycles of 1 min denaturation at 94 °C, 30 s annealing at 57 °C and 40 s extension at 72 °C. Reactions were finished by 7 min incubation at 72 °C. PCR products were examined for quality on ethidium bromide-stained agarose gels. PCR products were directly sequenced on ABI377 or ABI3700 sequencers (Biosciences, WUR) using the dideoxy chain-termination method and ABI PRISM Reaction Kit. Forward amplification primers were used as sequencing primers. To obtain phased haplotypes PCR products of eight genotypes of the GWDex7 amplicon and six of the GWD56 amplicon were cloned in pGEM-Teasy vector (Promega) and sequenced. On average twelve cloned PCR products were sequenced for each GWD haplotype to obtain a consensus sequence.

### Sequence variant detection and analysis

Alignment and quality scoring was done using the Staden software package (Staden 1996). Sequence variations (SNPs and short Indels) were detected using NovoSNP (Weckx et al. 2005). The allele copy number of SNPs was scored using both the Data Acquisition & Data Analysis software DAx7.1 (Van Mierlo Software Consultancy) and manual scoring. For nucleotide diversity and phylogenetic analysis the consensus haplotype sequences were compared with one another and with *S. lycopersicum*-derived sequences using MEGA 4 (Tamura et al. 2007) and TREECON (Van de Peer and De Wachter 1994) software. Similarity between each pair of sequences was calculated on the basis of percentage identity and tree construction was performed using the Neighbor-joining method. To estimate gene frequencies a program for the analysis of autotetraploid genotypic data, AUTOTET (Thrall and Young 2000) was used. The following statistics were calculated to describe the levels of genetic diversity: *Ai*, the average number of alleles per individual at a locus; *Ho*, the observed heterozygosity; and *He*, the expected heterozygosity. In order to compare the genotype proportions with those expected under Hardy–Weinberg equilibrium the mean fixation index (*F*) was calculated and the chi-squared (*χ*^2^) test was used to evaluate deviations of *F* from zero. Pedigree information was collected from the potato pedigree database (Van Berloo et al. 2007) and inspected for abnormalities in *StGWD* allele transmission using Pajek (De Nooy et al. 2005) and Cytoscape (Shannon et al. 2003).

### High resolution melting analysis

Amplicons for HRM genotyping were generated from 15 ng genomic DNA template. PCR amplifications were performed in 10 μl reactions using 2 μl of F-524 Phire™ 5 × reaction buffer (Finnzymes), 0.1 μl Phire™ Hot Start DNA Polymerase (Finnzymes), 1 μl LCGreen™ Plus + (BioChem) and 0.25 μl of 5 mM primers. PCR and heteroduplex formation were performed using the following conditions: 94 °C, 2 min; 40 cycles, 94 °C, 5 s; fragment-dependent Tm, 10 s; 72 °C, 10 s; a denaturation step of 30 s at 94 °C and renaturation by cooling to 30 °C. Amplicons were genotyped using the LightScanner® System (Idaho Technology).

### Phenotypic data collection

The tetraploid genotypes of this study were grown in two years and starch was isolated from both years. The phosphate content of starch was however analyzed for only one year and for a subset of 207 genotypes, because the assay is laborious. Starch phosphate measurements of individual samples were repeated in triplicate. For this measurement, approximately 20 mg starch (dry weight) was added to 250 μl 70% HClO_4_ and heated at 250 °C for 25 min. 50 μl 30% H_2_O_2_ was added and the mixture was heated at 250 °C for another 5 min. After cooling down the volume was increased to 2 ml by adding H_2_O. 100 μl of the sample was pipetted into a 96-well microtiter plate and 200 μl of color reagent (0.75% (NH_4_)6Mo_7_O_24_·4H_2_O, 3% FeSO_4_·7H_2_O and 0.75% SDS dissolved in 0.375 M H_2_SO_4_) was added. After incubation for 10 min at room temperature, the absorbance was measured at 750 nm, and compared to the absorption of a calibration curve to determine the sample PO_4_ concentration in nmol PO_4_/mg starch.

### Association analysis

For the analysis of phenotypic data and marker-trait association SPSS (IBM) was used. The association analysis was performed using a linear mixed model. The multivariate model was arranged to simultaneously assess the significance of all haplotype effects. Copy number of each of the haplotypes were modeled as fixed effects and haplotypes A_1_ to A_5_ were modeled as nested factors of grouped haplotype A. Variance components were estimated by the REML method. A general linear model was applied to estimate the explained phenotypic variance of associated haplotypes. For this, copy number of the haplotype within each clone was tested separately.

## Results

### Sequence diversity and haplotype analysis

**Fig. 1:**
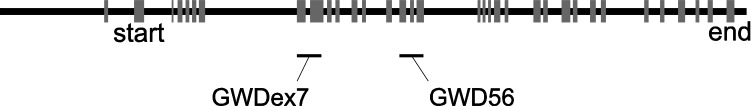
Gene model of *StGWD* genomic sequence. The 19,188 bp genomic sequence from genotype RH89-039-16 contains 34 exons (grey boxes) and 4392 bp of coding sequence. The GWDex7 (627 bp) and the GWD56 (606 bp) amplicons used for re-sequencing and genotyping are indicated

A panel of five monoploid and nine diploid potato accessions was selected to gain an initial insight into *StGWD* nucleotide polymorphism among *S. tuberosum* clones. Seven PCR amplicons were Sanger sequenced and assessed for single locus amplification, SNPs and Indels. Amplicons derived from the monoploid accessions had sequence chromatogram peaks representing a single haplotype. Amplicons of diploid accessions displayed double chromatogram peaks at discrete nucleotide positions as expected for heterozygous accessions. Of the seven different amplicons, three amplicons showed no indel polymorphisms. Indel polymorphisms can result in undecipherable sequence chromatograms. Two amplicons were selected to identify SNPs and haplotypes in a broader panel of 430 tetraploid potato cultivar and progenitor lines. The GWDex7 amplicon (627 bp) includes a large part of the gene region from exon 8 to exon 9. The GWD56 amplicon (606 bp) covers exon 15 to exon 17 (Fig. 1).

For the tetraploid accessions, high quality sequence chromatograms with an average read length of 523 bp were generated for 398 cultivars. A small number of accessions showed low quality chromatograms in repetitive runs. In the approximately 1 kb of accessible DNA sequence of the two amplicons, 81 polymorphisms were detected and quantitatively scored. Four of these polymorphisms were of multi-allelic (three tri-allelic, one tetra-allelic) nature and 77 of bi-allelic nature. The average number of polymorphic sites—which ignores the fact that polymorphisms co-segregate in haplotype blocks—was 1 polymorphism/12 bp.

Using the sequence information of both monoploid and diploid accessions seven initial haplotypes were inferred (A_1_, A_2_, A_3_, B, C, D, F). Three haplotypes (A_1_, A_2_, B) were observed among the five monoploid accessions. Haplotypes of the diploids were deduced by subtracting already identified haplotypes from the sequence chromatograms. Haplotypes of the tetraploid potato germplasm collection could not be directly inferred from the unphased sequence chromatograms due to the highly heterozygous state and high SNP frequency. Putative haplotype models for these accessions were deduced by identifying sets of co-segregating SNPs. For this we calculated the squared correlation coefficient (*r*^*2*^) between the copy numbers of all polymorphisms. Co-segregating polymorphisms were assigned to putative haplotypes and novel haplotypes were identified by sequencing cloned amplicons of a number of corresponding potato accessions. All polymorphisms were assigned to 16 verified haplotypes, and haplotype-specific tag SNPs could be identified (Table 1).

The quantitatively scored tag SNPs were used to assign an allele copy number and genotype composition to each cultivar. In case haplotypes contained multiple tag SNPs tagging the same haplotype the best quantifiable SNP was selected for copy number estimate. When a haplotype contained no unique tag SNP the allele copy number was inferred by subtracting the copy number of already tagged allele(s) from the “tag” SNP shared by the alleles (Table 2). For the GWDex7 amplicon identical-in-state haplotypes A_2_, A_3_, A_4_ and A_5_ and haplotype D were multi-marker defined. For the GWD56 amplicon haplotypes A_1_ and A_2_ were identical-in-state and there were four multi-marker defined haplotypes. The allele copy numbers found for the haplotypes of the GWDex7 amplicon invariably matched the allele copy numbers in the GWD56 amplicon. Using the selected tag SNPs it was possible to assign a four-allele genotype to 384 (96%) of the tetraploid potato cultivars (Supplementary Fig. 1).

**Fig. 2:**
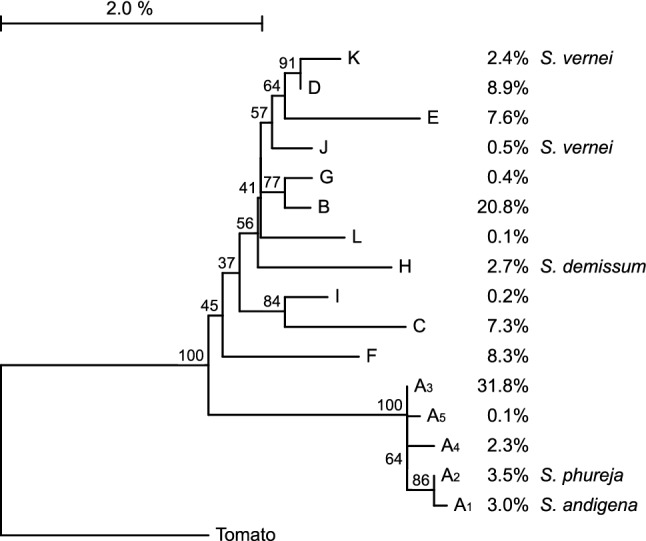
Dendogram of the 16 *GWD* haplotypes. The distances were computed using the Jukes-Cantor method and the tree inferred using the Neighbor-joining method. The percentage of replicate trees in which the associated haplotypes clustered together in the bootstrap test (1000 replicates) are shown next to the branches. For each allele the frequency and—when identified—the source is given. The tomato (*S. lycopersicum* cv. Heinz) sequence was used as out-group to root the tree

To investigate the sequence similarity between the detected haplotypes, a Neighbor-joining dendrogram was constructed using amplicon sequences of *S. lycopersicum* as out-group (Fig. 2). Over the 1 kb of DNA sequence of the two amplicons, the tomato haplotype was to a high degree similar (95.4–96.5%) to the haplotypes observed in the potato germplasm set. Sequence similarity between the 16 potato haplotypes ranged from 96.8 to 99.9%. Distance between the two most distant potato haplotypes (A_1_ and E) approached the sequence divergence observed between potato and tomato.

The estimated nucleotide diversity between the 16 potato haplotypes was π = 18.5 × 10^−3^ and translated into an average SNP diversity of ≈ 1 SNP/54 bp (1/π). At the protein level, the analyzed haplotypes included 302 codons of five exons. Of those codons 36 showed polymorphisms, causing 24 non-synonymous changes and 15 synonymous changes. No well-defined dysfunctional mutations such as stop codon, splicing site or frame shift mutations were found. For estimates of nucleotide diversity at the population level, the frequencies at which haplotypes occurred was considered. Six haplotypes had an allele frequency above 5%, and 10 had a frequency below 5% (Table 3). In the sampled population of 398 cultivars, we found a population frequency adjusted nucleotide diversity value of π = 16.2 × 10^−3^. Between two randomly selected homologues alleles, this translated into ≈ 1 SNP/62 bp.

### Pedigree analysis

To verify that the identified haplotypes were identical-by-descent and to identify putative sources of the haplotypes we performed a pedigree analysis. For 218 fully genotyped cultivars at least one parental cultivar had also been genotyped, and for 56 of these both parents were genotyped. For 22 out of the 218 genotyped parents/offspring pairs a mismatch was observed. In 12 occasions the mismatch repeatedly involved the parental genotypes of the cultivars AM 78-3704, Sirtema, Early Rose and Patersons Victoria. For several of the alleles, the putative source of the allele was found (Table 3). Haplotypes A_1_, H, I, J and K were found to be relatively new in the analyzed genepool. Haplotype G was found only in five heirloom potato cultivars. Other haplotypes were present in both ancient and new potato cultivars.

### Genetic diversity

Gallais (2003) proposed the following terms to describe tetraploid genotypes: monogenic (aaaa), digenic-simplex (aaab), digenic-duplex (aabb), trigenic (aabc) and tetragenic (abcd), terms we adopt here. When for a cultivar only one of the GWD amplicons was successfully re-sequenced, the five haplotypes that were identical-in-state in either of the amplicons (A_1_, A_2_, A_3_, A_4_ and A_5_) could not always be fully resolved. To strengthen the analysis of genotypic variation, these similar haplotypes were grouped into a single haplotype A group. A monogenic condition was observed in nine cultivars that were monogenic for haplotype A. Four of these contained only the major haplotype A_3_ and were truly homozygous at the *StGWD* locus. The five other cultivars contained three copies of the A_3_ allele and a copy of either the A_1_ or A_2_ allele. All other cultivars were heterozygous. We observed 77 tetragenic, 185 trigenic, 76 digenic-simplex and 37 digenic-duplex cultivars. The average number of alleles per individual (*Ai*) was 2.86 when the haplotypes A were grouped and estimated at 3.08 when using all 16 haplotypes. A total of 111 different genotypic classes were observed. The number of cultivars per class ranged from 1 to 27 (3.5 cultivars per class on average). The most abundant genotypic class was AAAB occurring in 27 cultivars, followed by AABB, AABD and AABF. Observed and expected heterozygosity (*Ho* = 0.765, *He* = 0.758) were in close agreement when assuming random chromosome segregation. A χ^2^ test showed that the mean fixation index (F) was in accordance with Hardy–Weinberg expectations.

### Associations with starch phosphate content

**Fig. 3:**
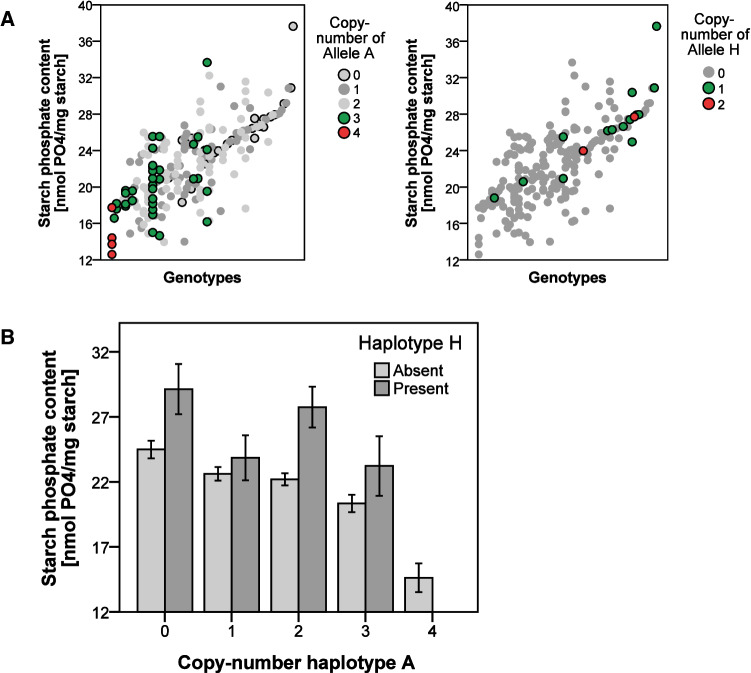
**A** Association between *GWD* allele-copy number and starch phosphate content for haplotypes A and H. Cultivars with the same four-allele genotype (80 different four-allele genotypes were found in 203 cultivars with measured starch phosphate content) have the same x-coordinate. Cultivars with different genotypes are ordered according to the increasing average starch phosphate content of the genotype. **B** Combined bar plot of the association between haplotypes A and H and the starch phosphate content of cultivars. Error bars show the standard error of the mean

Starch phosphate content was measured for 203 of the 398 genotyped cultivars. It ranged from 12.6 to 37.7 nmol PO_4_/mg starch, with an average of 22.5 ± 4.3 nmol PO_4_/mg starch. Variation in starch phosphate content within the 80 genotypic classes with measured starch phosphate contents was substantial. Two genotypic classes differed significantly from the other classes. Average starch phosphate content of homozygous class AAAA (14.6 nmol PO_4_/mg starch, n = 4) was significantly lower than the other classes. The single cultivar representing class BBCH had a significantly higher starch phosphate content (37.7 nmol PO_4_/mg starch). Linear mixed model analysis, modeling all haplotypes, identified significant independent associations to starch phosphate content for the grouped haplotype A (p-value 0.009) and haplotype H (p-value 0.015). The haplotype A association explained approximately 13.4% of the populations phenotypic variance and showed a negative association with starch phosphate content. Haplotype H showed a positive association and explained around 4.7% of the variance (Fig. 3).

### Validation in segregating populations

**Fig. 4:**
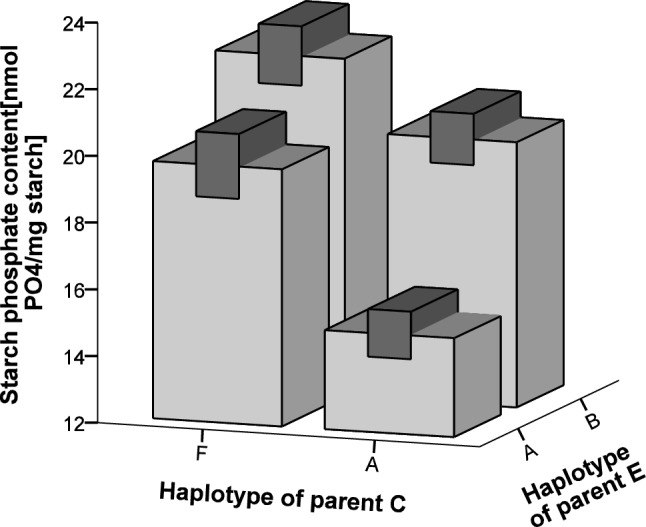
Amount of phosphorylated starch in the four genotypic *GWD* classes of the C × E population. Similar to the results found for the association analysis, the C × E mapping population plants lacking allele A have significantly higher starch phosphate content while the offspring homozygous for allele A have significant lower starch phosphate content. Error bars (dark grey) show the standard error of the mean

To confirm the association of the haplotype A, the GWD genotypes of 93 plants of the diploid potato C × E mapping population were resolved using HRM. Three distinct GWD haplotypes were observed in the parental genotypes. Haplotype A_2_ is shared between both parents, haplotype F is unique to the C-parent and haplotype B unique to the E-parent. Similar to the results found for the association analysis, the C × E mapping population plants lacking allele A had significant higher starch phosphate content while the offspring homozygous for allele A had significant lower starch phosphate content (Fig. 4).

**Fig. 5:**
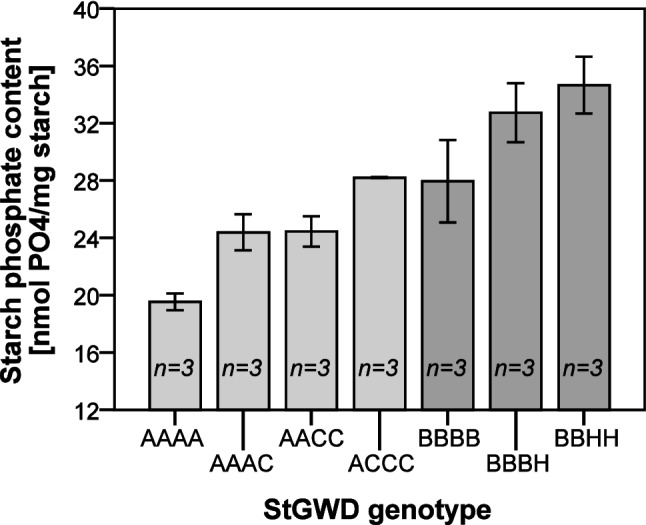
Starch phosphate content observed in descendants from a cross between Astarte (AACI) × Voran (AACC) (light grey bars), as well as descendants of selfed Sunrise (BBHH) (dark grey bars). Error bars show the standard error of the mean

To verify the association of the haplotype A and H we also genotyped 76 tetraploid offspring of a cross Astarte (A_3_A_3_CI) × Voran (A_3_A_3_CC) and 34 tetraploid offspring of a selfing population of Sunrise (BBHH) using HMR. In a study by Noda et al. (2004), who measured starch phosphorous content in six potato cultivars, a low phosphorous content was reported for cultivar Astarte. Starch phosphate content was measured in 34 offspring of the Astarte × Voran cross and in 19 offspring of Sunrise. For the Sunrise offspring we only obtained offspring genotypes with either no, one or two copies of the H allele. For the Astarte × Voran cross we analyzed only those plants that had allele A_3_ and/or C. Offspring with allele H showed a clear tendency (p value 0.070) towards higher starch phosphate content and offspring homozygous for allele A_3_ had a significant (p value < 0.001) lower phosphate content (Fig. 5).

## Discussion

### Nucleotide diversity

Two regions of the *StGWD* gene of 627 bp and 606 bp were analyzed by direct sequencing of PCR products from monoploid, diploid and tetraploid potato clones. Analysis of the sequence chromatograms, along with verification of a number of haplotypes using cloned PCR products, allowed us to identify a set of 16 haplotypes and their tag SNPs. The tag SNPs were instrumental to fully genotype 384 tetraploid potato cultivars and to study the genetic variation and phenotypic effect of the *StGWD* gene.

DNA sequence variation in potato is exceptionally abundant. We found an overall frequency of polymorphic sites of one variant every 12 bp for the *StGWD* gene. For this large panel of cultivars and progenitor lines, the number of polymorphic sites even exceeds the level of one variant per 21–23 bp found in previous studies (Rickert et al. 2003; Simko et al. 2006). The frequency of polymorphic sites and the molecular diversity can however vary widely, depending on how many clones, which regions and which genotypes are being analyzed. The study of Simko et al. (2006) involved 47 samples, including some wild accessions, and was re-sequenced for 66 loci. Comparison of nucleotide diversity between Simko et al. (2006) and our study shows that this statistic is more stable across studies (π = 14.6 × 10^−3^ and π = 16.2 × 10^−3^, respectively).

As expected, the average within-potato sequence diversity in our study does not exceed the nucleotide diversity between Solanum species at the *StGWD* locus. However, the level of sequence homology of the two most distant potato haplotypes approaches the tomato/potato species sequence divergence. Considering tomato/potato divergence time of 7.3 MYA (Wang et al. 2008), most observed haplotypes seem to have diverged long time ago, at least predating domestication in potato. Some haplotypes were observed only in recent cultivars and likely resulted from efforts to introduce late blight and nematode resistance from related species (Bradshaw and Ramsay 2005). Analyzed cultivars harboring haplotype H have a lineage descending from clone USDA 96-56. It is likely that this haplotype has been introduced into the *S. tuberosum* genepool together with the introduction of the chromosome 5 *Phytophthora infestans R1* resistance gene from *S. demissum* (Sinden and Sanford 1981). For another haplotype, haplotype A_1_ it is likely that it has been introgressed into the cultivated genepool from the CPC 1673 donor of the chromosome 5 potato cyst nematode (*Globodera rostochiensis*) resistance allele *H1* (Ellenby 1952). And haplotype K seems to be introduced into the genepool by introgression of the chromosome 5 *Globodera pallida* nematode resistance from clone VTN 62-33-3 (Ross and Hunnius 1986).

### Genetic diversity

The haplotype tag SNPs, with a quantitative scoring of allele copy number for two independent amplicons, gave us the possibility to exploit the full genotypic information. We used this genotypic information to evaluate the genetic diversity in the analyzed set of cultivars and accessions. Intra-individual heterozygosity (*Ho* = 0.77) and the mean observed number of haplotypes per plant (*Ai* ≈ 3.1) are high at the *StGWD* locus. Both the number of haplotypes and heterozygosity are markedly higher than those reported in an earlier allozyme study of 13 loci in tetraploid potato cultivars (Oliver and Martínez Zapater 1984), which seems to demonstrate the superior resolution of SNP markers compared to allozyme studies. The number of observed haplotypes was also higher than those reported in more recent potato re-sequencing studies (Li et al. 2005; Sattarzadeh et al. 2006; Simko et al. 2004) and comparable to a multi-locus study employing SSR markers on the same cultivar set (D'hoop et al. 2010). Furthermore, the above average levels of alleles and heterozygosity observed in individual potato cultivars suggest that an underestimation of heterozygosity caused by allele homoplasy should be of a minor magnitude and strengthen our conclusion that the alleles are identical-by-descent. Full allelic resolution of the *StGWD* locus would however require the complete gene to be re-sequenced, while in this study we only sequenced parts of the gene. Therefore, it cannot be excluded that currently unresolved haplotypes, identical-in-state to the identified haplotypes, remain in the genepool. Near complete re-sequencing of the *StGWD* gene in 84 potato cultivars and accessions using massively parallel sequencing has however not identified new alleles of the *StGWD* gene (Uitdewilligen et al. 2013).

### Association analysis of *StGWD* haplotypes with starch phosphate content

In potato there is only a small diversification into subpopulations. This diversification is along cultivars used for fresh consumption, processing (chips, fries), and potatoes used for the starch industry (D'hoop et al. 2010). We did not observe differences in *StGWD* allele frequencies in these subpopulations and therefore omitted correction for population structure in the association analysis.

Starch phosphate content is hardly influenced by environmental conditions (Haase and Plate 1996; Werij et al. 2012) and can be measured with a small technical error (Noda et al. 2006). A large variation in starch phosphate content within each genotypic class of GWD alleles was however observed in the tetraploid association mapping panel. This large variation indicates that, similar to the diploid C × E mapping population, in tetraploid cultivars multiple QTL with major effects on different genomic locations are associated with the trait. In fact the involvement of GWD2, Soluble Starch Synthase III and Branching Enzyme 1 and 2 has been shown by Carpenter et al. (2015).

Starch phosphate measurements and QTL analysis in the diploid C × E potato mapping population has been described previously (Werij et al. 2012). The QTL analysis showed three major additive QTLs on chromosomes 2, 5 and 9, each explaining approximately 20% of the observed variance. The QTL on chromosome 5 co-localized with the *StGWD* locus, a key enzyme involved in starch phosphorylation (Zeeman et al. 2007). We re-sequenced the parental genotypes of the C × E population and identified the GWD haplotypes in the C × E offspring using HRM. The reducing effect of haplotype A on starch phosphate content detected by the association analysis, is confirmed in the C × E population. Additionally, we verified the phenotypic effect of haplotype A and haplotype H in two tetraploid cross populations. Whether the association between starch phosphate content and these haplotypes is caused by a difference in RNA expression level or functional variation in the StGWD protein needs to be determined by further study.

Results from the present study indicate that a haplotype association analysis approach is a robust tool for mapping quantitative loci with relatively strong effects in commercially important potato populations, even without considering population structure. The fact that only a small proportion of the explained variance can be captured in this way is clear but also that this has already profound effects on the total phosphate content. Whether the observed effects connected to allele A (decrease) or allele H (increase) are sufficient to merit breeding efforts on starch phosphate content for commercial purposes remains to be evaluated but it is evident that even for these types of quantitative traits in a tetraploid background specific effects of individual alleles can contribute to the final phenotype and can be used as breeding targets.

**Table 1:**
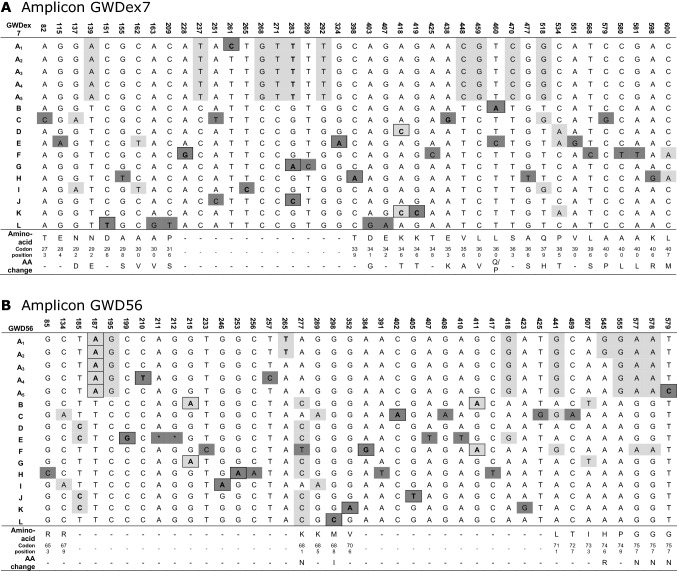
Phased *GWD* haplotypes for amplicon (A) GWDex7 and (B) GWD56

Positions are relative to the start of the amplicon. Haplotype-defining tag SNPs are color-coded; Dark grey bases indicate SNPs which tag a single haplotype, light grey bases indicate SNPs shared by multiple haplotypes. Deletions in haplotypes are shown as asterisks. The last three lines in the tables indicate the amino acids and their codon position in the reference sequence and non-synonymous changes. The non-coding SNPs in introns are indicated by missing codon positions (-). Tag SNPs used for copy number estimation are shown in bold

**Table 2:** *GWD* haplotype tag SNPs

	Amplicon tag SNP	
GWD Haplotype	GWDex7	GWD56
A_1_	SNP261C	SNP265T
A_2_	SNP283T—Allele A_1_	SNP265T
A_3_	SNP283T—Allele A_1_	SNP187A—Allel A_4_—Allel A_5_
A_4_	SNP283T—Allele A_1_	SNP210T
A_5_	SNP283T—Allele A_1_	SNP579C
B	SNP460A	SNP215A—Allel F
C	SNP438G	SNP402A
D	SNP418C—Allele K	SNP185C—Allel E—Allel J—Allel K
E	SNP324A	SNP199G
F	SNP228G	SNP384G
G	SNP283A	SNP215A—Allel B
H	SNP398A	SNP253A
I	SNP265C	SNP246A
J	SNP283C	SNP405T
K	SNP419C	SNP352A
L	SNP151T	SNP298C

All SNPs were quantitatively scored and used for copy number estimation, but only indicated SNPs were used to estimate haplotype copy number and to detect the full four-allele genotype of the tetraploid cultivars. For a cultivar re-sequenced successfully in only one amplicon, either GWDex7 or GWD56, the A haplotypes can be identical-in-state to each other. Some haplotypes are without unique haplotype tag SNP and are multi-marker defined. E.g. haplotype D in the GWDex7 amplicon is defined by SNP418C—Allele K (= SNP419C)

**Table 3:** Allele-frequencies of *GWD* haplotypes in the collection of ~ 400 sequenced tetraploid potato cultivars and breeding lines

GWD haplotype	Allele count	Allele frequency (%)	Possible sources
A_1_	46	3.00	Observed in descendants of *S. andigena* clone CPC 1673 used as donor of resistance against *Globodera rostochiensis*
A_2_	49	3.50	Observed in *S. phureja* genotype DM1-3 516R44
A_3_	464	31.80	
A_4_	33	2.30	
A_5_	2	0.10	Observed only in Lenape and Golden Wonder
B	331	20.80	
C	117	7.30	
D	139	8.90	
E	121	7.60	
F	129	8.30	
G	6	0.40	Observed in heirloom cultivars
H	43	2.70	Observed in progeny of *S. demissum* introgression clone USDA 96-56 used as donor for R1 resistance against *Phytophthora infestans*
I	3	0.20	Observed in Astarte and its descendants
J	8	0.50	Observed in progeny of *S. vernei* introgression clone VE 66-295
K	39	2.40	Observed in progeny of *S. vernei* introgression clone VTN 62-33-3, donor of resistance against *Globodera pallida* Pa2
L	1	0.10	Observed only in Hindenburg

Five haplotypes have an allele frequency below 1% and only six haplotypes have an allele frequency above 5% (major alleles). The haplotype A group contains the minor alleles A_1_, A_2_, A_4_ and A_5_ and the common allele A_3_. By examining potato pedigree data the putative donor of some of the minor alleles is identified

## Supplementary Information

Below is the link to the electronic supplementary material.

Supplementary file1 (DOCX 30 kb)

## Data Availability

All data is enclosed either in main text or as supplementary data. Other data can be requested from the corresponding author.
