# Supplementary material for: Allelic variation for alpha-Glucan Water Dikinase is associated with starch phosphate content in tetraploid potato
Source: Plant Mol Biol. 2022 Jan 7;108(4-5):469–80. doi: 10.1007/s11103-021-01236-7 (PMC8894227; doi:10.1007/s11103-021-01236-7)
Supplement: Supplementary file 1 — Supplementary file1 (DOCX 30 kb) [file 11103_2021_1236_MOESM1_ESM.docx]

**Supplementary figures and tables Genetic regulation of starch phosphate content in potato**

**Supplementary table 1. List of primers used for sequencing and genotyping *StGWD*.**

| Primer name | Forward | Reverse | Optimum Tm |
| --- | --- | --- | --- |
| GWDex7 | GGAATATGAGGCTGCTCGAACT | TCTGCTCCTCCTTCTCCTTGGC | 56°C |
| GWD56 | TGAAATAAGCAAGGCTCAGGAC | ATAGTGACCTAAATCACGCAAA | 55°C |
| GWD_G1 | TCTTTGAACAGCTAGCAGAAAA | GCAGCTCTTTAACCAAAATG | 57°C |
| GWD_G2 | AACCAGGAAGTAGGAACCAG | CACACTCCCATCTCATGTTG | 57°C |
| GWD_G34 | AACAACCATCCAAACAAGGT | CCAGGACTTTTGGATAATGC | 57°C |
| GWD_G5 | TGTTCGACTGTGGACAAAAC | AGGGTTGCTATGTGAATGGT | 57°C |
| GWD_G6 | TAATGGTGATCCATTTGCAG | CAGAGAGTGCGAGATTTTCA | 57°C |
| GWD_G7 | TCAAGCTCTTTCAATGTCCA | AATCCTTCCTTCCTTTGCTT | 57°C |
| GWD_end_2 | GGATGAGGAGGAAAAAGTTG | TGCAATACATAATGCGTGTG | 57°C |
| GWD_HRM_#1 | CTTGAGCTTGAGAAAGGCAT | CAAAGTCTCTCTTCTTTCTTTGGAT | 60°C |


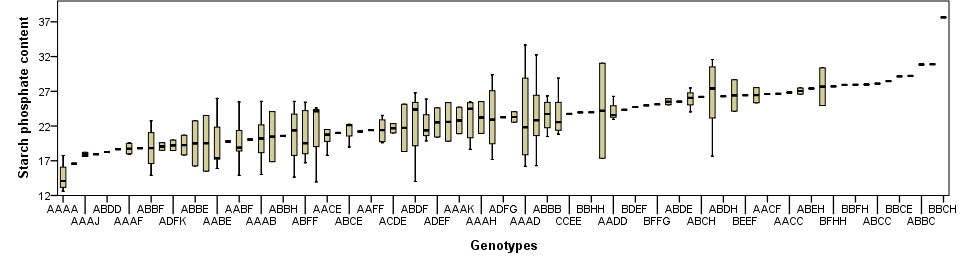


**Figure S1.** Association between GWD genotype and starch phosphate content. Cultivars with the same four-allele genotype (80 different four-allele genotypes were found in 400 cultivars) have the same x-coordinate and are represented as boxplots or as a small single stripe in case the genotype is represented by a single cultivar. Genotypes are ordered according to the average starch phosphate content. The four low phosphate composition cultivars with genotypes AAAA and the single high phosphate cultivar with genotype BBCH differ significantly for starch phosphate content from the genotypes with intermediate phosphate content.
